# Independent Risk Factors for Deaths due to AIDS in Chongqing, China: Does Age Matter?

**DOI:** 10.3389/fmed.2020.586390

**Published:** 2021-02-17

**Authors:** Yihong Zhou, Zhongping Yang, Min Liu, Yanqiu Lu, Yuanyuan Qin, Xiaoqing He, Yanming Zeng, Vijay Harypursat, Yaokai Chen

**Affiliations:** ^1^Division of Infectious Diseases, Chongqing Public Health Medical Center, Chongqing, China; ^2^National Key Laboratory for Infectious Diseases Prevention and Treatment With Traditional Chinese Medicine, Chongqing Public Health Medical Center, Chongqing, China

**Keywords:** aging, HIV, AIDS, death, opportunistic infections

## Abstract

**Background:** People living with HIV (PLWH) are aging worldwide, and different management strategies may be required for older and younger PLWH. However, demographic characteristics, illness distribution, mortality, and independent risk factors in the PLWH population in China are not yet fully understood, especially in patients aged 50 years or older.

**Methods:** We conducted a retrospective analysis of 4445 HIV-positive Chinese inpatients in Chongqing, China.

**Results:** The mortality rate in patients 50 years or older (the older group) was significantly higher than that in those under 50 years (the younger group) (*p* < 0.001). In the younger group, independent risk factors for death included: nadir CD4+ T-cell counts <200 cells/μL, not owning medical healthcare insurance, not being on cART, injection drug use, and having one of the following comorbidities: Pneumocystis pneumonia, cryptococcal meningitis, AIDS malignancy, non-AIDS malignancy, and kidney disease. In the older group, independent predictors of death included: being urban residents, nadir CD4+ T-cell counts <200 cells/μL, not being on cART, and having comorbidities such as Pneumocystis pneumonia, hepatitis C, talaromycosis, non-AIDS malignancy, and kidney disease.

**Conclusions:** Demographic characteristics, illness distribution, mortality, and independent risk factors for death in HIV-positive patients differ between the older group and the younger group, indicating that a changing suite of medical and allied support services may be required the for management of older PLWH.

## Introduction

Since the introduction of combination antiretroviral therapy (cART), the life expectancy of people living with HIV (PLWH) has gradually been approaching that of the general population (1, 2). This improvement in life expectancy and longevity has resulted in an increase in the number of PLWH living into older age, with an estimated 4 million of PLWH being over 50 years old globally. This number has doubled since the introduction of cART in 1996, and continues to increase (3, 4). Also, an increasing proportion of new HIV infections in older adults are contributing to this increase in PLWH aged 50 or more (5–7). This trend is global, and is observable in both high-income, and low- and middle-income countries (LMIC). In high-income countries, half of PLWH are now aged 50 years or older (8, 9). In the United States, for example, the median age of HIV-infected adults has passed 50 years, with Canada, Australia, and most European countries closely following (10). In LMIC, more PLWH are surviving into older age, with a dramatic increase in the number of older PLWH (11). In Latin America, the Caribbean, Sub-Saharan Africa, Asia, the Middle East, and North Africa, emerging trends regarding PLWH survival and aging are similar to that in high-income countries (10). In mainland China, the number of new Acquired Immune Deficiency Syndrome (AIDS) cases among over-50-year-olds is increasing year on year. The number of PLWH in China aged 50 and above in 2014 was 4.2 times the number in 2008 (27,520 vs. 6,599) (12). The proportion of over-50-year-olds increased from 13.7% in 2008 to 26.6% in 2014, representing a 2-fold increase (12–14).

Compared with younger PLWH, older PLWH are more likely to have different demographic, clinical, hematological, and immunological baseline homeostatic conditions, which are likely to affect outcomes of disease (10, 15, 16). Older PLWH face a variety of unique challenges including possible accelerated aging, and higher incident rates of non-AIDS-related co-morbidities, such as stroke, hypertension, heart disease, liver cirrhosis, dyslipidemia, diabetes, kidney diseases, and non-AIDS malignancy (8, 11, 17). In addition, older PLWH face more challenges across stages of the HIV care continuum due to a more rapid decline in immune function (18), and higher mortality rates (7, 11, 16, 19). In high-income settings, the contribution of non-AIDS-related diseases to mortality is increasing in PLWH, particularly in older people (20–23), whereas in sub-Saharan Africa, infectious diseases, especially tuberculosis, fungal, and bacterial infections, remain the main causes of morbidity and mortality (24, 25).

Chongqing is the largest centrally controlled municipality in China, consisting of 26 districts, eight counties, and four autonomous counties, and is spread over an area of 82,402 square kilometers. It is also the most populous Chinese municipality, having a population of over 30 million. Between 2007 and 2012, the number of reported HIV infections in this province grew at an average annual rate of 19.7%, which is substantially higher than the national rate (3.1%) (26). The average proportion of patients who were 50 years or older at HIV diagnosis increased dramatically from 3.6% between 1988 and 2003 to 45.4% between 2013 and 2017 (16). Chongqing is an essential regional hub of south-western China and is more heavily affected by the HIV/AIDS epidemic compared with other regions in China. However, the demographic characteristics, prevalence of comorbidities, and predictors of death in the PLWH population in this region are not fully understood, especially in those who are 50 years or older. In order to investigate the differences between PLWH aged 50 or older and PLWH aged <50 years, we conducted a retrospective analysis on HIV infected patients hospitalized at Chongqing Public Health Medical Center from January 2014 to January 2018, aiming to provide key information about the PLWH in this region, and to improve clinical management and outcomes for older PLWH. This analysis might serve as a template to guide healthcare policy aimed at better meeting the complex healthcare needs of the rapidly increasing number of older PLWH in China.

## Materials and Methods

### Study Design and Setting

This was a retrospective cohort study of opportunistic infections and non-AIDS co-morbidities among hospitalized PLWH from January 2014 to January 2018. All serologically confirmed HIV-infected patients that were admitted to Chongqing Public Health Medical Center during the study period were enrolled in this study. Where a particular patient was admitted more than once during the study period, these admissions were collated and recorded as one admission, and all clinical laboratory data and diagnoses were also collated as data for the single patient. Unique patient identification numbers were generated to distinguish re-admissions and follow-up visits. In our analysis, we only used data collected from patients entered into the electronic medical record system (27). All eligible hospitalized patients were stratified into two study groups: the older group, consisting of PLWH aged 50 years or older, and the younger group, consisting of PLWH <50 years old.

Our study was conducted in accordance with the Declaration of Helsinki, and prior approval was obtained from the institutional review board of Chongqing Public Health Medical Center. The institutional review board waived the requirement for written informed consent, since this study was retrospective in nature, and all patient data were analyzed in anonymity.

### Disease Diagnosis

For our study, AIDS malignancy was defined as non-Hodgkin lymphoma, cervical premalignant lesions, and Kaposi sarcoma. Malignancies other than non-Hodgkin lymphoma, cervical premalignant lesions, and Kaposi sarcoma were defined as non-AIDS malignancy. Heart disease included the following diagnoses: myocardial infarction, angina pectoris, coronary artery bypass grafting, and angioplasty. Diagnostic criteria for hypertension, diabetes, and kidney disease included two or more consecutive measurements of blood pressure measurements >140/90 mm Hg, fasting serum glucose levels >126 mg/dL, and estimated glomerular filtration rate (eGFR) <60 mL/min by use of the modification of diet in kidney disease estimating equation, respectively. All patients were tested for syphilis, hepatitis C, hepatitis B, and fasting lipids, and diagnoses were confirmed according to testing results. A large fraction of the diagnoses of Pneumocystis pneumonia, oral candidiasis, and toxoplasma encephalopathy were presumptive due to easier establishment of a clinical diagnosis and limited availability of pathogenetic testing for these specific diseases.

### Statistical Analysis

Patient information was anonymized and de-identified prior to analysis. The following variables were extracted from the medical records of Chongqing Public Health Medical Center: age, gender, medical healthcare insurance, urban clinic location, nadir CD4+ T-cell count, injection drug use, cART initiation, and diagnoses. For deceased patients, duration of hospitalization was recorded and causes of death were extracted from death certificates. For discharged patients, duration of hospitalization and diagnoses on discharge were extracted from the discharge certificate of the medical records. All analyses were performed using SPSS software Version 18.0 (IBM-SPSS, Chicago, IL, USA). Differences in proportions were compared by the Chi-squared test or Fisher's Exact test as appropriate. *P* < 0.05 were considered statistically significant. Strength of association was determined by calculating adjusted odds ratios (aORs) and their 95% confidence intervals (CIs). In identifying independent factors associated with deaths, variables were initially analyzed using a bivariate model, and subsequently independent risk factors were identified by means of a logistic regression model using a forward, stepwise approach, which began with inclusion of all variables associated with deaths on bivariate analysis (*p* ≤ 0.1), and subsequently included only those variables with *p* ≤ 0.05 in the final model. The sensitivity, specificity, area under the receiver operating characteristic curve, Nagelkerke *R*^2^, and Hosmer-Lemeshow tests were used to assess the quality of the logistic regression model.

## Results

### Study Flow and Characteristics of the Study Population

From January 2014 to January 2018, there were 6,065 AIDS-associated admissions to Chongqing Public Health Medical Center. After collation of multi-admissions and exclusion of cases with incomplete data and length of hospital stay ≤ 24 h, 4,445 hospitalized patients with AIDS were enrolled in this study. These patients were stratified into an older group (50 years or older), and a younger group (<50 years old). Among these 4,445 patients, 1,866 (42.0%) were 50 years or older and 2579 (58.0%) were <50 years old.

Among the data of 4,445 patients eligible for analysis, 78.6% were male and 21.4% were female; 89.2% were married, and 93.7% owned medical healthcare insurance (Table 1).

**Table 1:** Baseline characteristics of 4,445 hospitalized PLWH, stratified by age.

**Variable**	**Total**	**The younger group (*n* = 2,664, 58.2%)**	**The older group (*n* = 1,917, 41.8%)**	***p***
**SEX**				
Male	3,493	2,045 (79.3%)	1,448 (77.6%)	0.174
Female	952	534 (20.7%)	418 (22.4%)	
**MARRIED**				
YES	3,963	2,121 (82.2%)	1,893 (98.7%)	<0.001
NO	482	458 (17.8%)	24 (1.3%)	
**MEDICAL HEALTHCARE INSURANCE**				
YES	4,167	2,354 (91.3%)	1,813 (97.2%)	<0.001
NO	278	225 (8.7%)	53 (2.8%)	
**URBAN RESIDENTS**				
NO	526	358 (13.9%)	168 (9.0%)	<0.001
YES	3,737	2,121 (82.2%)	1,616 (86.6%)	
Unknown	182	100 (3.9%)	82 (4.4%)	
**NADIR CD4 COUNT PER mm3**				
<200 cells/μL	3,441	1,969 (76.3%)	1,472 (78.9%)	0.133
≥200 cells/μL	1,001	608 (23.6%)	393 (21.1%)	
Unknown	3	2 (0.1%)	1 (0.1%)	
**INJECTION DRUG USE**				
NO	4,394	2,534 (98.3%)	1,860 (99.7%)	<0.001
YES	51	45 (1.7%)	6 (0.3%)	
**On-Cart**				
NO	2,824	1,512 (58.6%)	1,312 (70.3%)	<0.001
YES	1,621	1,067 (41.4%)	557 (29.7%)	

*cART, Combination antiretroviral therapy*.

Compared with the younger group, more patients in the older group were married (98.7 vs. 82.2%, *p* < 0.001), had medical healthcare insurance (97.2 vs. 91.3%, *p* < 0.001), and were urban residents (86.6 vs. 82.2%, *p* < 0.001), and fewer patients in the older group had a history of injection drug use (0.3 vs. 1.7%, *p* < 0.001), or had received antiretroviral treatment (29.7 vs. 41.4%, *p* < 0.001) (Table 1).

### Difference in the Prevalence of Diseases Between the Two Groups

Among the 4,445 patients, 3,852 (86.7%) were admitted to hospital due to the presence of an overt opportunistic infection. There was no statistical difference in the prevalence of opportunistic infections between the older group and the younger group (86.2 vs. 87.0%, *p* = 0.471). Compared with those in the younger group, patients in the older group had a significantly lower prevalence of tuberculosis, syphilis, hepatitis C, cryptococcal meningitis, toxoplasma encephalopathy, and talaromycosis. In addition, patients in the older group had a significantly higher prevalence of Pneumocystis pneumonia, non-AIDS malignancy, stroke, heart disease, diabetes, hypertension, and kidney disease, compared with those in the younger group (Table 2).

**Table 2:** Distribution of opportunistic infections and non-AIDS co-morbidities in 4,445 hospitalized PLWH stratified by age.

**Diagnosis**	**Younger group**	**Older group**	***p***
**OPPORTUNISTIC INFECTION**			
Oral candidiasis	1,311 (50.8%)	941 (50.4%)	0.790
Tuberculosis	1,068 (41.4%)	680 (36.4%)	0.001
Pneumocystis pneumonia	637 (24.7%)	516 (27.7%)	0.027
Cytomegalovirus infection	493 (19.1%)	346 (18.5%)	0.630
Syphilis	185 (7.2%)	59 (3.2%)	<0.001
Hepatitis C	183 (7.1%)	41 (2.2%)	<0.001
Hepatitis B	219 (8.5%)	153 (8.2%)	0.728
Cryptococcosis meningitis	110 (4.3%)	52 (2.8%)	0.009
AIDS malignancy	17 (0.7%)	22 (1.2%)	0.067
Toxoplasma encephalopathy	48 (1.9%)	19 (1.0%)	0.023
Talaromycosis	49 (1.9%)	8 (0.4%)	<0.001
**NON AIDS CO-MORBIDITIES**			
Non-AIDS malignancy	42 (1.6%)	93 (5.0%)	<0.001
Stroke	76 (3.0%)	162 (8.7%)	<0.001
Heart disease	51 (2.0%)	159 (8.5%)	<0.001
Diabetes	43 (1.7%)	146 (7.8%)	<0.001
Liver cirrhosis	42 (1.6%)	37 (2.0%)	0.378
Hypertension	34 (1.3%)	155 (8.3%)	<0.001
Dyslipidemia	27 (1.0%)	17 (0.9%)	0.652
Kidney disease	256 (9.2%)	308 (16.5%)	<0.001

*AIDS, Acquired Immune Deficiency Syndrome*.

### Difference in Mortality Between the Two Groups

The overall mortality rate for 4,445 hospitalized PLWH was 9.2%. Mortality of patients in the older group was significantly higher compared to the younger group, viz. 11.3 and 7.7%, respectively (*p* < 0.001). Table 3 shows the distribution of opportunistic infections and non-AIDS comorbidities in the two groups. Compared with the younger group, patients who died during hospitalization in the older group had a higher prevalence of oral candidiasis, tuberculosis, Pneumocystis pneumonia, cytomegalovirus infection, and hepatitis C.

**Table 3:** Distribution of mortality in two age groups stratified by opportunistic infections and non-AIDS comorbidities.

	**Total**	**Younger group**	**Older group**	***p***
Total	409 (9.2%)	198 (7.7%)	211 (11.3%)	<0.001
**OPPORTUNISTIC INFECTION**				
Oral candidiasis	221 (9.8%)	113 (8.6%)	108 (11.5%)	0.025
Tuberculosis	159(9.1%)	84 (7.9%)	75 (11.0%)	0.025
Pneumocystis pneumonia	154 (13.4%)	71 (11.1%)	83 (16.1%)	0.014
Cytomegalovirus infection	100 (11.9%)	49 (9.9%)	51 (14.7%)	0.035
Syphilis	14 (5.7%)	9 (4.9%)	5 (8.5%)	0.299
Hepatitis C	28 (12.5%)	17 (9.3%)	11 (26.8%)	0.002
Hepatitis B	28 (7.5%)	16 (7.3%)	12(7.8%)	0.847
Cryptococcal Meningitis	22 (13.6%)	14 (12.7%)	8 (15.4%)	0.645
AIDS malignancy	8 (20.5%)	4 (23.5%)	4 (18.2%)	0.682
Toxoplasma encephalopathy	12 (17.9%)	7 (14.6%)	5 (26.3%)	0.438
Talaromycosis	11 (19.3%)	7 (14.3%)	4 (50.0%)	0.059
**NON AIDS CO-MORBIDITIES**				
Non-AIDS malignancy	25 (18.5%)	8 (19.0%)	17 (18.3%)	0.915
Stroke	25 (10.5%)	15 (10.6%)	10 (10.4%)	0.971
Heart disease	30 (14.3%)	7 (13.7%)	23 (14.5%)	0.895
Diabetes	23 (12.2%)	4 (9.3%)	19 (13.0%)	0.513
Liver cirrhosis	10 (12.7%)	3 (7.1%)	7 (18.9%)	0.218
Hypertension	19 (10.1%)	4 (11.8%)	15 (9.7%)	0.959
Dyslipidemia	1 (2.3%)	1 (3.7%)	0 (0.0%)	1.000
Kidney disease	102 (18.8%)	39 (16.5%)	63 (20.5%)	0.245

*AIDS, Acquired Immune Deficiency Syndrome*.

### Independent Risks of Death

For the entire cohort, we conducted a multivariate logistic regression analysis of factors that may have had an impact on the risk of death. All variables with a *p* ≤ 0.1 in the univariate analysis were included in a logistic regression model using a forward stepwise approach, and adjusted for age (years), urban clinic location, nadir CD4+ T-cell count, whether on- cART, injection drug use, and having one of the following comorbidities: Pneumocystis pneumonia, Cytomegalovirus disease, syphilis, hepatitis C, cryptococcal meningitis, AIDS malignancy, toxoplasma encephalopathy, talaromycosis, non-AIDS malignancy, stroke, heart disease, and kidney disease. We observed that the following factors had significantly increased the risk of death in our hospitalized patients: being aged 50 years or older, being urban residents, nadir CD4+ T-cell counts being <200 cells/μL, not being on cART, being injection drug users, and having one of the following comorbidities: Pneumocystis pneumonia, cryptococcal meningitis, AIDS-related malignancy, toxoplasma encephalopathy, talaromycosis, non-AIDS malignancy, and kidney disease (Table 4). The−2Loglikelihood of the logistic regression was 2499.863, with a correct classification rate of 90.8% (Hosmer-Lemeshow test *p* = 0.879 and Nagelkerke's *R*^2^ = 0.105). The area under the receiver operating characteristic curve was 0.713. We selected the numerical value with the highest Youden index {(sensitivity+specificity)-1} as our cut-off point for the predicted probability (cut-off for probability = 9.05%) (Table 4).

**Table 4:** Independent predictors of mortality in hospitalized PLWH.

	**Total**	**Younger group**	**Older group**
	**Adjusted OR(95% CI)**	**Adjusted OR(95% CI)**	**Adjusted OR(95% CI)**
**AGE (YEARS)**			
<50 years	1		
≥50 years	1.295 (1.043, 1.608)^*^		
**URBAN CLINIC LOCATION**			
NO	1		1
YES	1.301 (0.912, 1.855)		2.217 (1.137, 4.324)^*^
Unknown	2.365 (1.382, 4.046)^**^		4.864 (2.086, 11.339)^***^
**NADIR CD4 COUNT**			
CD4 <200/mL	1	1	1
CD4≥200/mL	0.442 (0. 311, 0. 629)^***^	0.404(0. 241, 0. 677)^**^	0.490(0. 305, 0.787)^**^
**MEDICAL HEALTHCARE INSURANCE**			
YES		1	
NO		1.653 (1.038, 2.631)^*^	
**On-cART**			
NO	1	1	1
YES	0.480 (0. 372, 0. 621)^***^	0.386 (0.269,0.554)^***^	0.599(0.415,0.863)^**^
**INJECTION DRUG ABUSE**			
NO	1	1	
YES	2.842 (1.328, 6.083)^**^	2.791 (1.188,6.558)^*^	
**PNEUMOCYSTIS PNEUMONIA**			
NO	1	1	1
YES	1.890 (1.507, 2.370)^***^	1.816 (1.313,2.510)^***^	2.012 (1.464, 2.764)^***^
**HEPATITIS C**			
NO			1
YES			3.353 (1.593, 7.056)^**^
**CRYPTOCOCCAL MENINGITIS**			
NO	1	1	
YES	1.706 (1.055, 2.760)^*^	1.980 (1.080,3.633)^*^	
**AIDS MALIGNANCY**			
NO	1	1	
YES	3.940(1.737, 8.937)^**^	6.120 (1.845,20.304)^**^	
**TOXOPLASMA ENCEPHALOPATHY**			
NO	1		
YES	1.959 (1.006, 3.818)^*^		
**TALAROMYCOSIS**			
NO	1		1
YES	2.410 (1.198, 4.846)^*^		9.928 (2.267, 43.487)^**^
**NON-AIDS MALIGNANCY**			
NO	1	1	1
YES	2.226 (1.389, 3.567)^**^	2.921 (1.191, 6.090) ^*^	2.111 (1.191, 3.741)^*^
**KIDNEY DISEASE**			
NO	1	1	1
YES	2.659 (2.059, 3.433)^***^	2.731 (1.845, 4.043)^***^	2.549 (1.816, 3.578)^***^
Area under the receiver operating characteristic curve	0.713	0.717	0.708
Sensitivity	0.614	0.596	0.659
Specificity	0.710	0.724	0.688
Nagelkerke *R*^2^	0.105	0.104	0.105
Hosmer-Lemeshow *P* value	0.879	0.999	0.938

* *p < 0.05*,

** *p < 0.01*,

*** *p < 0.001*.

*cART, Combination antiretroviral therapy; AIDS, Acquired Immune Deficiency Syndrome; OR indicates odds ratio. CI indicates confidence interval*.

In the younger group, we conducted a multivariate logistic regression analysis of factors that may have had an impact on the risk of death. All variables with a *p* ≤ 0.1 in the univariate analysis were included in a logistic regression model using a forward stepwise approach, and adjusted for nadir CD4+ T-cell count, possession of medical healthcare insurance, on-cART, injection drug use, and having one of the following comorbidities: oral candidiasis, Pneumocystis pneumonia, Cytomegalovirus disease, cryptococcal meningitis, AIDS malignancy, toxoplasma encephalopathy, talaromycosis, non-AIDS malignancy, and kidney disease. The factors that independently significantly increased the risk of death were nadir CD4+ T-cell counts being <200 cells/μL, not having medical healthcare insurance, not being on cART, injection drug use, and having one of the following comorbidities: Pneumocystis pneumonia, cryptococcal meningitis, an AIDS malignancy, a non-AIDS malignancy, and kidney disease. The−2Loglikelihood of the logistic regression was 1282.751, with a correct classification rate of 92.4% (Hosmer-Lemeshow test *p* = 0.999 and Nagelkerke's *R*^2^ = 0.104). The area under the receiver operating characteristic curve was 0.717. We selected the numerical value with the highest Youden index as our cut-off point for the predicted probability (cut-off for probability = 7.7%) (Table 4).

In the older group, we conducted a multivariate logistic regression analysis of factors that may have had an impact on the risk of death. All variables with a *p* ≤ 0.1 in the univariate analysis were included in a logistic regression model using a forward stepwise approach, and were adjusted for urban residents, nadir CD4+ T-cell count, whether on-cART, and having one of the following comorbidities: Pneumocystis pneumonia, Cytomegalovirus disease, hepatitis C, toxoplasma encephalopathy, talaromycosis, non-AIDS malignancy, and kidney disease. Independent risk factors for death included: being urban residents, nadir CD4+ T-cell count being <200 cells/μL, not being on cART, and having comorbidities such as Pneumocystis pneumonia, hepatitis C, talaromycosis, non-AIDS malignancy, and kidney disease. The−2Loglikelihood of the logistic regression was 1215.188, with a correct classification rate of 88.6% (Hosmer-Lemeshow test *p*=0.938 and Nagelkerke's *R*^2^ = 0.105). The area under the receiver operating characteristic curve was 0.708. We selected the numerical value with the highest Youden index as our cut-off point for the predicted probability (cut-off for probability = 10.3%) (Table 4).

The common risk factors in the two groups were nadir CD4+ T-cell counts, whether on-cART, and having one of the following comorbidities: Pneumocystis pneumonia, non-AIDS malignancy, and kidney disease. However, urban clinic location, hepatitis C, and talaromycosis were risk factors applicable to just the older group (Table 4).

## Discussion

Chongqing Public Health Medical Center, the largest government-funded tertiary referral infectious disease hospital in south-western China, serves the municipality of Chongqing and surrounding provinces, and has had over 2000 HIV-associated medical admissions annually for the past several years.

In the USA, the overall rate of AIDS-related illnesses decreased between 2001 and 2008 (35). Reductions in AIDS-related infections were reported over a similar period in patients in Brazil and France (36), and in children in the USA (37). In our study, opportunistic infections remained the leading causes of hospital admission (86.7%) regardless of age, which is similar to global data (23). This finding may partly be explained by the low nadir CD4+ T-cell counts and the low cART coverage in our cohort of patients, less than half of whom were receiving cART at admission. This explanation is particularly true of patients in the older group, with over 70% of these patients not being on cART, and nearly 80% having nadir CD4+ T-cell counts <200 cells/μL. As shown in Table 1, the proportion of on-cART patients in the older group was also significantly lower, indicating that increased cART coverage and cART adherence support are highly warranted in this population.

In older individuals, delay in HIV diagnosis is not unusual in Chongqing, primarily due to unwillingness in this population to be tested, and difficulty in identifying individuals who do not have commonly recognized risk factors for infection, who thus avoid or bypass routine screening tests. An encouraging social factor, however, is that the majority of those aged 50 years or older had medical healthcare insurance in Chongqing, which makes it feasible to integrate health examinations and HIV screening tests in this population. If this group has access to early diagnosis and treatment, their overall disease situation would be greatly improved, since cART use has been demonstrated to be associated with improved survival, increased CD4+ T-cell counts, and reduced numbers of HIV-associated hospital admissions (23, 38).

Table 2 shows that compared with the younger group, patients in the older group were more likely to have Pneumocystis pneumonia, a non-AIDS malignancy, stroke, heart disease, diabetes, hypertension, and kidney disease, and were less likely to have tuberculosis, syphilis, hepatitis C, cryptococcal meningitis, toxoplasma encephalopathy, and talaromycosis. This observation suggests that different patterns of medical and allied support services may be needed for different age groups. For example, opioid substitution therapy, and hepatitis C screening and treatment are more applicable to PLWH <50 years of age, since a larger proportion of this population have a history of injection drug use. This is contrary to observations made in New York City (39). Sexually transmitted disease (STD) screening also needs to be enhanced because of the higher prevalence of syphilis in this population. Unfortunately, the older cohort in our study experiences a double burden of disease, having a higher prevalence of both communicable diseases and non-communicable chronic diseases, according to our results. This finding indicates that medical services based on a multidisciplinary team may be indicated for these older patients, since a larger proportion in this cohort have strokes, heart disease, diabetes, hypertension, and kidney disease, and some general physicians may not be well-enough equipped with the specialist knowledge and skills required to adequately and comprehensively manage other communicable and non-communicable chronic diseases and conditions occurring in tandem with overt HIV infection and AIDS.

Our results showed that the overall mortality in our hospitalized patients was 9.2%, which is lower than that in Malawi (22.7%) (11), and in Bangladesh (19.5%) (28). We also found that similar to Malawi (11) and other low-income countries (28, 29), older patients in our cohort had higher mortality rates than younger adults (*p* < 0.001), and most deaths were associated with opportunistic infections. This is in contrast to reports from high–income countries (30, 31), where deaths in HIV-infected patients occur mainly secondary to non-AIDS-related medical conditions.

It is true that older people have higher mortality than younger ones. However, the reasons of mortality in older people living with HIV may differ with HIV-uninfected older people, and thus, it is necessary to understand risk factors for death in HIV-infected older individuals and provide targeted evidence for healthcare professionals (32). In our study, we not only found that the older people have higher mortality than younger ones, but also found that demographic characteristics, illness distribution, mortality, and independent risk factors for death differ between the older group and the younger group.

Overall, our results showed that the prevalence of opportunistic infections was higher in the younger group, whereas the prevalence of non-AIDS malignancy, stroke, heart disease, diabetes, hypertension, and kidney disease was higher in the older group. Our study results also showed that factors such as being aged 50 years or older, being urban residents, nadir CD4+ T-cell counts being <200 cells/μL, not being on cART, being injection drug users, and having one of the following comorbidities: Pneumocystis pneumonia, hepatitis C, talaromycosis, non-AIDS malignancy, and kidney disease were independent risk factors for death. Among these factors, and concordant with observations in Bangladesh (28), patients in the older group had a significantly higher prevalence of, and mortality from, Pneumocystis pneumonia compared with the younger group, indicating that Pneumocystis pneumonia prophylaxis awareness, delivery, and utilization should be a priority in older PLWH.

We stratified the entire cohort by age and found that older patients and younger patients had common, as well as differing risk factors for mortality. As shown in Table 4, we found that both groups shared common risk factors for death, and these factors included nadir CD4+ T-cell counts, whether on-cART, and having one of the following comorbidities: Pneumocystis pneumonia, non-AIDS malignancy, and kidney disease. However, urban residential status, presence of co-infection with hepatitis C, and talaromycosis comorbidity in the older group bestowed significantly higher mortality risks, whereas in patients in the younger group, a concomitant diagnosis of cryptococcal meningitis imparted significantly higher mortality risk. The most likely explanation for this is that cryptococcal meningitis was more likely to occur in younger patients, and the elderly were not predisposed to cryptococcal meningitis, mostly because of older Chongqing patient's limited environmental exposure (33).

Interestingly, older patients who were urban residents tended to have a higher mortality in our cohort. The most likely explanation for this is that older people may be more willing to choose small clinics within their urban neighborhood for examination, assessment, and treatment due to factors related to patient convenience. In addition, more older rural patients may choose staying at home when becoming seriously ill, because dying at home is considered to be psychologically more auspicious and comfortable for patients facing an impending death, since it gives family members and friends more time to be with the person, and grants them more autonomy, privacy, and dignity (34). If this is indeed the case, it would thus be more difficult for healthcare providers and researchers to collect and collate information related to these deaths.

It is possible, also, that attending doctors at the small clinics attended by older patients lack specialist training in HIV-associated illnesses, and thereby may miss diagnoses, or diagnose HIV-associated illnesses much later in the course of these diseases. In contrast, younger people are likely to be more willing to travel longer distances to access specialized HIV clinics and tertiary level care, in order to seek more comprehensive medical attention and care.

There are limitations to this study. Firstly, this was a retrospective, observational study, and study data may have been subject to incorrect interpretation, and other general limitations related to retrospective observational investigations. Secondly, data for some variables were incomplete, and this may result in a degree of bias. Thirdly, although the diagnoses of most diseases were definitive, a small number of diagnoses in our study were presumptive. Fourthly, sample size was also limited as we have analyzed only 409 cases of death due to AIDS out of 4,445 hospitalized PLWH. Finally, this report reflects the experience of just one specialist hospital that primarily provides care for Chinese patients with HIV, and therefore the results may not be generalizable to all HIV-infected patients admitted to other hospitals in China, or to different populations elsewhere in the world.

In conclusion, this study observed that opportunistic infections remain the dominant reason for admission to hospital in south western China, and the demographic characteristics, illness distribution, mortality, and independent risk factors for death differ between patients aged 50 years or older, and those under 50 years. Our age-based analysis demonstrates the evolution and complexity of health needs of older PLWH in China. Our results suggest that a changing set of medical and allied support services may be required for older PLWH, such as cART adherence support, a requirement for specialist physicians trained in both HIV-related diseases and non-HIV related chronic diseases, enhanced national screening services, and targeted early intervention programs.

## Data Availability Statement

The datasets generated for this study are available on request to the corresponding author.

## Ethics Statement

Our study was conducted in accordance with the Declaration of Helsinki and prior approval was obtained from the institutional review board of Chongqing Public Health Medical Center. The institutional review board waived the requirement for written informed consent, since this study was retrospective and all patient data were analyzed in anonymity.

## Author Contributions

YZ: patients' enrollment, data generation and collection, statistical analysis, and writing of the manuscript. ZY: data generation and collection, statistical analysis, and writing of the manuscript. ML: patient enrollment, data generation, and analysis of data. YL: analysis of the data and writing of the manuscript. YQ: data generation and collection. XH: patient enrollment, data generation and collection. YZ: patient enrollment, data generation and collection. VH: manuscript revision. YC: study design, clinical data discussion, and manuscript revision. All authors approved the final version of the manuscript.

## Conflict of Interest

The authors declare that the research was conducted in the absence of any commercial or financial relationships that could be construed as a potential conflict of interest.

## Abbreviations

AIDS: Acquired Immune Deficiency Syndrome
cART: Combination antiretroviral therapy
PLWH: people living with HIV
STDs: Sexually transmitted diseases
eGFR: estimated glomerular filtration rate
aORs: adjusted odds ratios
CIs: confidence intervals.
